# Interface Compositions as Determinants of Resveratrol Stability in Nanoemulsion Delivery Systems

**DOI:** 10.3390/foods9101394

**Published:** 2020-10-02

**Authors:** Adela Mora-Gutierrez, Rahmat Attaie, Maryuri T. Núñez de González, Yoonsung Jung, Sixto A. Marquez

**Affiliations:** 1 Cooperative Agricultural Research Center, Prairie View A&M University, Prairie View, TX 77446, USA; rattaie@pvamu.edu (R.A.); mtnunez@pvamu.edu (M.T.N.d.G.); yojung@pvamu.edu (Y.J.); 2 Department of Horticultural Sciences, Texas A&M University, College Station, TX 77843, USA; sixto46@tamu.edu

**Keywords:** resveratrol, casein, bovine, caprine, polysorbate-20, nanoemulsion delivery systems

## Abstract

The incorporation of hydrophobic ingredients, such as resveratrol (a fat-soluble phytochemical), in nanoemulsions can increase the water solubility and stability of these hydrophobic ingredients. The nanodelivery of resveratrol can result in a marked improvement in the bioavailability of this health-promoting ingredient. The current study hypothesized that resveratrol can bind to caprine casein, which may result in the preservation of the biological properties of resveratrol. The fluorescence spectra provided proof of this complex formation by demonstrating that resveratrol binds to caprine casein in the vicinity of tryptophan amino acid residues. The caprine casein/resveratrol complex is stabilized by hydrophobic interactions and hydrogen bonds. Hence, to study the rate of resveratrol degradation during processing/storage, resveratrol losses were determined by reversed-phase high performance liquid chromatography (RP-HPLC) in nanoemulsions stabilized by bovine and caprine caseins individually and in combination with polysorbate-20. At 48 h oxidation, 88.33% and 89.08% was left of resveratrol in the nanoemulsions stabilized by caprine casein (α_s1_-I)/polysorbate-20 complex and caprine (α_s1_-II)/polysorbate-20 complex, while there was less resveratrol left in the nanoemulsions stabilized by bovine casein/polysorbate-20 complex, suggesting that oxygen degradation was involved. The findings of this study are crucial for the food industry since they imply the potential use of caprine casein/polysorbate-20 complex to preserve the biological properties of resveratrol.

## 1. Introduction

Resveratrol (*trans*-3, 4′, 5-trihydroxy stilbene), which is mainly found in grapes and red wine, is a phytochemical with several potent biological activities such as antioxidant, anti-inflammatory, cardio-protective, neuroprotective, chemo-preventive, and anti-aging [1,2,3,4]. Since resveratrol utilization in aqueous foods is limited by its poor bioavailability due, in large part, to its poor aqueous solubility, and to its tendency to be unstable due to auto-oxidation and photosensitivity [5,6], there is a need for developing nanoemulsion delivery systems to enhance the water solubility and stability of resveratrol [7].

Emulsion droplets are a hydrophobic domain readily dispersed in aqueous foods. The lipid phase serves as a reservoir for other hydrophobic ingredients (e.g., flavors, nutrients, phytochemicals) which provides some protection against losses by chemical reactions and can limit or control their release [8]. The stability of emulsions in complex liquid systems is critical in order to provide a homogeneous product with the desired visual appearance, mouthfeel, texture, and shelf-life stability [9]. Caseins (α_s1_-, α_s2_-, β-, κ-) are widely valued for their emulsifying properties. The stability behavior of oil-in-water emulsions based on caseins is related to their structure and mechanical properties of adsorbed layers at the surface of the droplets [10]. When surfactants are used together with casein, competitive adsorption of two components toward the interface occurs [11], thereby leading to some emulsion instability by reduced steric and electrostatic repulsion [12]. However, very low concentrations of surfactants improve the role of casein at the oil-in-water interface at acidic pH [13], and the pH of milk (6.67) [14]. The roles of α_s1_-, α_s2_-, β-, and κ-casein in the interaction of polysorbate-20 with bovine and caprine caseins at the oil–water interface in resveratrol-enriched nanoemulsions have prompted this investigation. Therefore, the purpose of this study was to prepare resveratrol-loaded nanoemulsions with bovine and caprine caseins individually and in combination with polysorbate-20 to evaluate their effects on the degradation of resveratrol during processing/storage. Spectroscopic methods were used to elucidate the interaction of polysorbate-20 with bovine and caprine caseins in solution prior to their use as emulsifiers (stabilizers) in resveratrol-loaded nanoemulsions.

## 2. Materials and Methods

### 2.1. Materials

Resveratrol (purity >99.0%), polysorbate-20, β-casein from bovine milk (purity >90%), ethanol (200 proof, purity >99.5%), dimethyl sulfoxide (DMSO), and thimerosal were purchased from Sigma-Aldrich (St. Louis, MO, USA) and Thermo Fisher Scientific (Waltham, MA, USA). Medium-chain triglycerides (Neobee^®^ M- 5) was kindly donated by Stepan Company (Northfield, IL, USA). The analytical grade extraction solvents and HPLC-grade solvents were purchased from Thermo Fisher Scientific. Deionized water, prepared by passing distilled water over a mixed bed of cation–anion exchangers, was used throughout this study.

### 2.2. Preparation of Bovine and Caprine Caseins

The caprine caseins characterized by a high content of α_s1_-casein (type I) and α_s1_-casein (type II) were obtained from a French Alpine goat and an Anglo-Nubian goat, respectively [15]. Bovine casein was obtained from the milk of a Jersey cow. Briefly, casein was prepared from skimmed milk by precipitating at pH 4.6 and 30 °C, neutralizing at pH 7.0, dialyzing against deionized water, and lyophilizing. The integrity of the samples was confirmed by sodium dodecyl sulfate-polyacrylamide gel electrophoresis (SDS-PAGE) and densitometry was used to assess the relative concentration of casein components [16].

### 2.3. Preparation of Caprine β-Casein

Caprine β-casein was prepared by urea fractionation [17] followed by column chromatography on DEAE-cellulose in urea [17]. Most of the caprine β-casein was eluted in two sequential peaks designated fraction I (tubes 29 thru 31) and fraction II (tubes 42 thru 46). These two fractions were found by SDS-PAGE and densitometry to contain 98 and 95% of caprine β-casein, respectively [16]. These electrophoretic peaks possibly correspond to the caprine β-casein variants A and C of Moatsou et al. [18].

### 2.4. Fluorescence Spectroscopy

The fluorescence studies were performed on a Shimadzu spectrofluorometer RF-5000 (Shimadzu Corporation, Columbia, MD, USA) at 25 °C, using microcells with a working volume of 250 µL and a path length of 5 mm. The bovine and caprine caseins (in 5 mM phosphate buffer, pH 7.0) were excited at 280 nm and their emission spectra were recorded individually and in combination with polysorbate-20 at 25 °C. Polysorbate-20 was added to the protein solution in the cuvette, mixed and spectra were recorded after standing for 2 min. The concentration of bovine casein or caprine casein was 0.1 mg/mL and polysorbate-20 was 1 µM.

To study the interaction of resveratrol with bovine and caprine caseins, a stock solution (1000 µM) of resveratrol was prepared by dissolving resveratrol in 1.25% (*v*/*v*) ethanol in water. This concentration of ethanol is not detrimental to protein structure [19]. The resveratrol (1000 µM) was dissolved by continuous stirring. Samples for fluorescence measurement were prepared by mixing required volumes of the stock solution of resveratrol (1000 µM) and casein (bovine vs. caprine) and water to make a final volume of 10 mL. Resveratrol (in 5 mM phosphate buffer, pH 7.0) was excited at its λ_max_ (adsorption) of 308 and the emission spectra were recorded between 360 and 580 nm at 25 °C. Emission spectra were recorded in the concentration range mentioned in the legends of figures.

### 2.5. Circular Dichroism (CD) Spectroscopy

Circular dichroism (CD) experiments were carried out with bovine β-casein and caprine β-casein (0.1 mg/mL) in 5 mM potassium phosphate buffer at pH 7.0. Polysorbate-20 was added to the bovine and caprine β-casein samples at concentrations of 1 µM, 10 µM, and 100 µM. Samples were filtered through 0.45-µm-pore filters into a jacketed 0.5-mm-pathlength cylindrical cell made of far-UV quartz. CD spectra were recorded on an AVIV 60DS spectropolarimeter (Aviv Associates, Lakewood, NJ, USA) calibrated with *d*-10-camphorsulfonic acid. Successive measurements in the far-UV (190–250 nm) were made at 25 °C. Background contributions from the buffer or polysorbate-20 were subtracted. Ellipticities (θ) are reported as deg cm^2^ dmol^−1^. Secondary structure was estimated using CAPITO (http://capito.nmr.leibniz-fli.de) [20].

### 2.6. Resveratrol Binding to Bovine and Caprine Caseins in Solution

To evaluate the ability of the bovine and caprine caseins to bind resveratrol, mixtures of the casein solutions (2% *w*/*w*) dissolved in 5 mM phosphate buffer (pH 7.0) solution and resveratrol dissolved in ethanol (1.25% *v*/*v*), at a concentration of 500 µg/mL in the final mixture, were mixed using a magnetic stirrer at low speed for 30 min at 25 °C. The samples were then vortexed and incubated for 5 days at 25 °C. After 5 days of incubation at 25 °C, samples were centrifuged at 10,000× *g* (Beckman Instruments) for 15 min and the supernatant (0.05 mL) was diluted in 3.95 mL of DMSO. The unbound resveratrol was quantified by measuring the solution absorbance at 309 using a UV/vis model DU-530 spectrophotometer (Beckman Instruments) [21]. A solution of bovine casein or caprine casein in DMSO without resveratrol was used as a blank. Resveratrol dissolved in DMSO was used to construct a calibration curve with concentrations ranging from 1 µg/mL to 1000 µg/mL. More than 98.5% of resveratrol was bound to the bovine and caprine caseins.

### 2.7. Preparation of Resveratrol-Loaded Nanoemulsions

The oil phase consisted of medium-chain triglycerides (MCT). The aqueous phase consisted of 2% (*w*/*w*) of bovine casein or caprine casein bound to resveratrol dissolved in a buffer solution (5 mM phosphate buffer, pH 7.0) containing an anti-microbial (1 mM *w*/*v*) thimerosal. The final resveratrol concentration of the initial nanoemulsions stabilized by bovine and caprine caseins was as follows: 493 µg/mL (bovine), 496 µg/mL (caprine α_s1_-I) and 497 µg/mL (caprine α_s1_-II).

Coarse emulsions were formed by homogenizing 5% (*w*/*w*) of the oil phase with 95% (*w*/*w*) of the aqueous phase for 5 min with a handheld homogenizer (Biospec Products, Inc., Bartlesville, OK, USA) at low speed. In emulsions with polysorbte-20, 1000 µM of polysorbate-20 was added to the water phase before emulsification. The coarse emulsion was then homogenized five times at 100 MPa through a high-pressure TC5 homogenizer (Stansted Fluid Power, Harlow, UK). The emulsions were transferred to 50-mL Pyrex amber bottles and kept at 25 °C for 48 h in darkness. The quantity of resveratrol in nanoemulsions was monitored by RP-HPLC at 6, 12, 24, and 48 h.

The concentrations of unabsorbed proteins (emulsifiers) in the aqueous phase at 6 h and 48 h of incubation were measured as follows: aliquots of emulsions were centrifuged at 13,000× *g* (Beckman Instruments) for 30 min to separate the aqueous phase from the oil droplets. The aqueous phase was collected and filtered through a 0.10 µm-cellulose acetate filter (Sterlitech Corporation, Kent, WA, USA) to remove the small oil droplets. The amount of unabsorbed proteins in the aqueous phase of the emulsions was determined according to the method of Markwell et al. [22].

### 2.8. Physical Characterization of Resveratrol-Loaded Nanoemulsions

The particle size of the oil droplets in the resveratrol-loaded emulsions was measured at 0 h and 48 h of storage (at 25 °C) after homogenization with a SALD-2101 laser diffraction particle analyzer (Shimadzu Corporation, Columbia, MD, USA). The charge of the oil droplets in the resveratrol-loaded emulsion (zeta potential, ζ, mV) was measured at 0 h and 48 h of storage (at 25 °C) with a Zetasizer Nano ZS (Malvern Instruments, Worcestershire, UK). Samples were diluted 100 times in 5 mM phosphate buffer at pH 7.0.

### 2.9. Quantification of Resveratrol in Nanoemulsions

The resveratrol contents of the nanoemulsions were determined using RP-HPLC. Five milliliters of nanoemulsion were deproteinized by mixing with 1 mL of 1 M HCl and then 10 mL of acetonitrile was added to the mixture followed by vortex-mixing for 1 min. The supernatant was filtered (Whatman #541, Whatman International Ltd., Maidstone, UK) and collecting approximately 7 mL of filtrate. An aliquot (1 mL) of filtrate was collected and passed through a 0.45 µm syringe filter into a 2 mL amber vial. The sample solution (10 µL) was injected into an HPLC system.

The HPLC (Waters Corporation, Milford, MA, USA) instrument was equipped with a 515 pump, Waters auto-sampler model 717, a Waters C18 column (reversed phase, 250 mm × 4.6 mm, I. D., 5 µm), and a UV-visible photodiode array detector model 2489. Empower 2 software was used for analysis. The mobile phase consisted of a mixture of methanol: 10 mM potassium dihydrogen phosphate buffer (pH 6.8): acetonitrile (63:30:7, *v*/*v*/*v*) at a constant flow rate of 1 mL/min at 25 °C. Chromatographic analysis was conducted in isocratic mode, and the detection was carried out at 306 nm. An injection volume of 10 µL was used for all standards.

The calibration curve was constructed using resveratrol (10 µg to 500 µg/mL) with a correlation coefficient of 0.999. The elution time of *trans*-resveratrol was 2.817 min. All samples and standards were protected from light to avoid photochemical isomerization of resveratrol from *trans* to *cis* form.

### 2.10. Statistical Analysis

All experiments were conducted in triplicate and the data were analyzed using SAS 9.4 program (SAS Institute Inc., Cary, NC, USA). All data analyses were conducted by the PROC MIXED procedure of SAS. Tukey’s test applied for multiple comparisons at a significance level of α = 0.05%. Comparisons between the treatments with the responses for particle size and zeta potential were based on complete randomized design and used one-way analysis of variance (ANOVA). The treatment comparison for resveratrol (%) was conducted on two factorial design for emulsifier and time and used two-way analysis of variance (ANOVA).

## 3. Results and Discussion

### 3.1. Characterization of Bovine and Caprine Caseins

Caprine milk contains the same four casein fractions that bovine milk has, i.e., α_s1_-, α_s2_-, β-, and κ-caseins [23], but the α_s1_-casein fraction has very large individual quantitative variations among animals, partly due to the occurrence of genetic polymorphism [24]. This polymorphism gives rise to great variations in α_s1_-casein content of caprine milks from about 25% in certain caprine milks to total lack of it in others. In bovine milk, the consistency of the ratio of caseins in forming the structure of micelles aids in good quality products, whereas the variability of the casein ratio (α_s1_- to α_s2_-) in particular affects the micelle structure, which may lead to caprine milk products of differing quality, especially cheeses.

The electrophoretic α_s1_-band of slower mobility (labeled as α_s1_-I) in Table 1 seems to correspond to the α_s1_-E protein. The electrophoretic band labeled as α_s1_-II in Table 1 possibly corresponds to the sum of contributions from α_s1_-A, B or C variants [25]. The SDS-PAGE findings regarding the genetic polymorphism in the caprine casein (α_s1_-I) and caprine casein (α_s1_-II) samples used in this study should be interpreted with caution. Hence, the bands labeled as α_s1_-I and α_s1_-II are regarded as a trunked version of α_s1_-casein (Table 1). Moreover, the β-casein content of caprine casein (α_s1_-I) and caprine casein (α_s1_-II) is markedly higher than that of bovine casein (Table 1). The high hydrophobic character of the β-casein fraction [26] makes the caprine casein α_s1_-I and caprine casein α_s1_-II suitable candidates as encapsulating agents for hydrophobic ingredients such as omega-3 fatty acids, carotenoids [27,28], and resveratrol (this study) in food emulsions.

### 3.2. Binding of Polysorbate-20 to Caprine Casein in Solution

The understanding of the fluorescence properties of tryptophan is vital for its use as an intrinsic probe of protein structure and dynamics [29,30]. The subtle changes in fluorescence that are associated with changes in protein environment are revealed by fluorescence spectroscopy. Tryptophan is one of the major chromophores among the naturally occurring amino acids and is responsible for the absorbance shown by proteins at 280 nm. Bovine casein contains five tryptophan residues, of which Trp-164 and Trp-199 locate at α_s1_-casein, Trp-109 and Trp-193 at α_s2_-casein, and Trp-143 locates at β-casein [26]. These tryptophan residues contribute mainly to the intrinsic fluorescence of bovine casein. In the case of caprine casein, amino acid sequences located in homologous positions in homologous caseins may be different. In this study, the existence of such differences in caprine casein from bovine casein is suggested but not proven [28].

**Figure 1:**
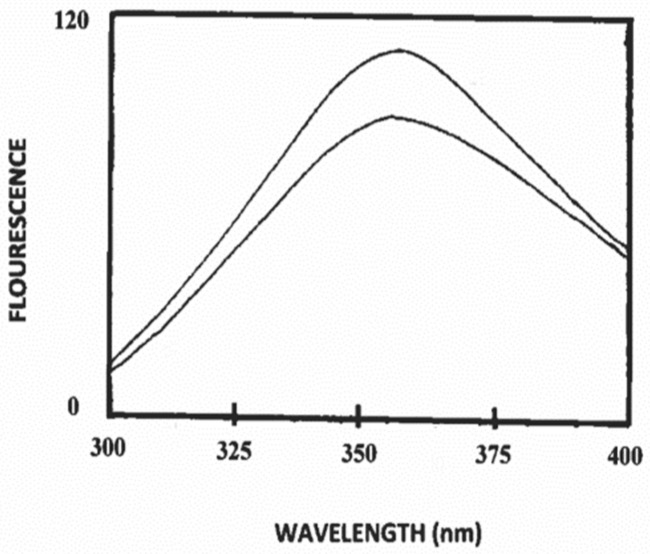
Fluorescence emission spectra (excitation wavelength 280 nm) of bovine casein alone and in combination with polysorbate-20 at 25 °C in 5 mM phosphate buffer, pH 7.0. Traces, from top to bottom, are bovine casein (0.1 mg/mL); and bovine casein (0.1 mg/mL) with added polysorbate-20 (1 µM).

The environment of tryptophan residues appears to be perturbed by the binding of polysorbate-20 to caprine casein (α_s1_-II) (Figure 1). A subtle shift in the wavelength of maximum emission (λ_max_), from 357 to 352 under solution conditions suggests that the tryptophan residues of caprine casein (α_s1_-II) were in a relatively more hydrophobic environment upon binding to polysorbate-20 (Figure 1). The intrinsic fluorescence intensity of caprine casein (α_s1_-II) decreased (Figure 1). The quenching of caprine casein (α_s1_-II) by polysorbate-20 is induced by complex formation (Figure 1). Polysorbate-20 seems to associate with caprine casein (α_s1_-II) and modify the surface of caprine casein (α_s1_-II), and the hydrophobic interior of caprine casein (α_s1_-II) could be made more accessible for polysorbate-20 [31]. These results reflect the hydrophobic tryptophan environment in the solvent accessible region of caprine casein/polysorbate-20 complex.

### 3.3. Estimation of Secondary Structure of β-Casein/Polysorbate-20 Complex in Solution

**Figure 2:**
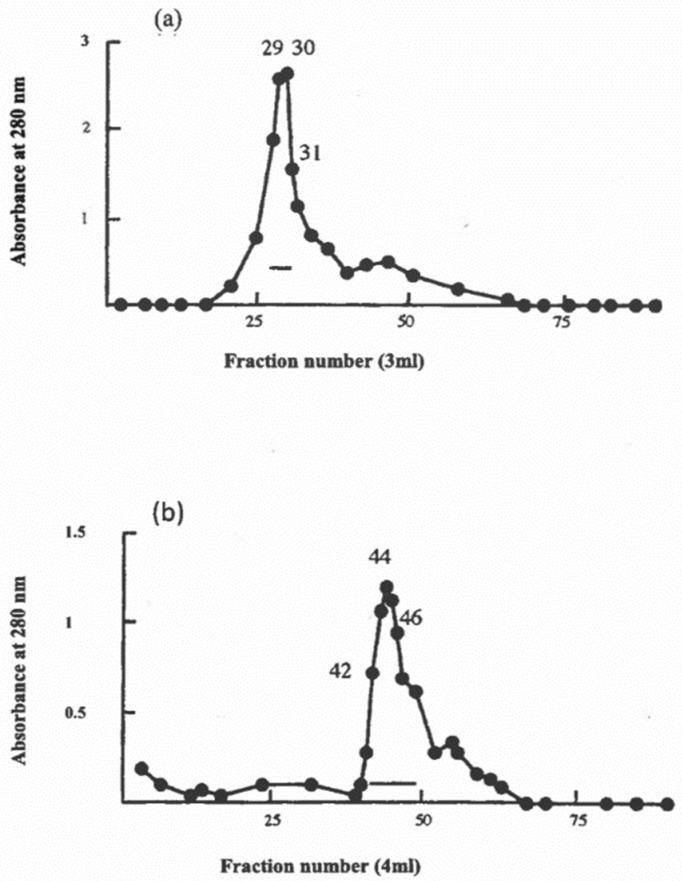
Fractionation of caprine β-casein by DEAE-cellulose chromatography. Fraction I (**a**) and Fraction II (**b**).

The high surface activity and bio-function of casein micelles from bovine milk are largely related to β-casein; thus, the study of the individual structural property of β-casein plays a key role in understanding the structure/function relationship of casein micelles from commercially important ruminant species [32]. The fractionation of β-casein from caprine casein is shown in Figure 2. To determine its potential for interacting with other components of the caprine casein micelles, the caprine β-casein fraction of Figure 2a was investigated by circular dichroism (CD) spectroscopy.

**Figure 3:**
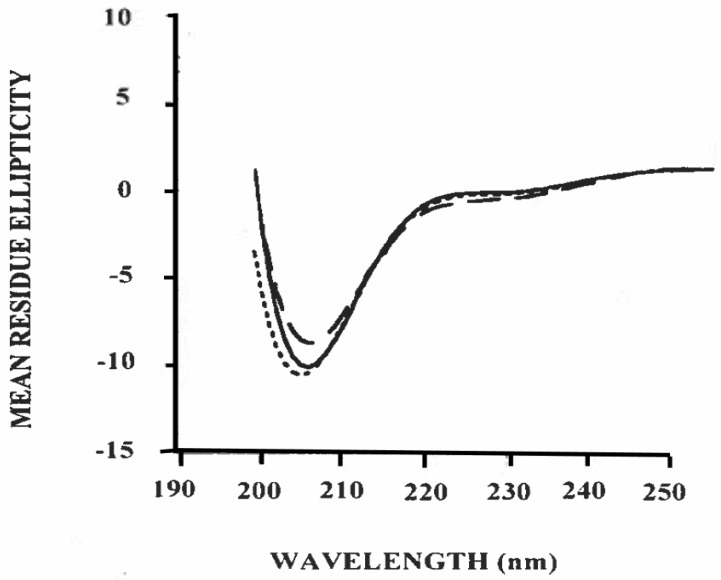
Circular dichroism spectra of caprine β-casein (0.1 mg/mL) in combination with polysorbate-20 at 25 °C in 5 mM phosphate buffer, pH 7.0. ― ― ― 1 µM polysorbate-20; ______10 µM polysorbate-20; - - - - 100 µM polysorbate-20.

Figure 3 shows the mean residue ellipticity (θ) as a function of wavelength in the far-UV region for the caprine β-casein/polysorbate-20 complex at 25 °C. The CAPITO analysis (Table 2) of the CD spectra shows a helical conformational stabilizing effect of polysorbate-20 on caprine β-casein. Hydrogen binding derived from hydroxyl groups of polysorbate-20 with amino acid residues of protein plays a key role in the formation of a hydrogen bond network for stabilizing the helical conformational change induced by surfactant, as previously observed in α-lactalbumin [33].

In the case of bovine β-casein, the secondary structure content showed 13.5% of α-helical, 17.9% of β-sheet, and 69.1% of irregular conformation (Table 2), indicating the intrinsically disordered structure of bovine β-casein [34]. Upon binding with polysorbate-20, the content of α-helical, β-sheet and irregular conformation was calculated as 16.8%, 21.0%, and 62.7%, respectively, suggesting that the α-helical and β-sheet conformation of bovine β-casein increased, but the irregular structure was reduced. The CD data suggest that the changes in the secondary structure of bovine β-casein induced by polysorbate-20 are related to the disordered regions and the hydrophobic domains of this protein [34]. The lauric acid chains of polysorbate-20 may be aligned along the α-helical and β-sheet structures in order to stabilize disordered regions and expose aromatic residues of bovine β-casein, thereby increasing the secondary structure of bovine β-casein [35]. The hydrophobic interactions and hydrogen bonds are the main factors governing the stabilization [33,34,36].

The data of Figure 3 show the potential of an α-helical-like structure for caprine casein (α_s1_-II) upon binding to polysorbate-20 (Table 2). Such structures could be important in the stabilization of hydrophobic β-casein aggregates. Previous work demonstrated that the stabilization of the hydrophobic region in α_s1_-casein–ĸ-casein interactions results in the ability of ĸ-casein to stabilize α_s1_-casein. Hydrophobic amino acids play an important role in casein–casein interactions and therefore determine, to some extent, a variety of milk product functional properties, such as foaming, gelation and emulsification. These results confirm that the complex milk protein system plays a central role in the formation of micellar casein in this system.

### 3.4. Binding of Resveratrol to Bovine and Caprine Caseins in Solution

**Figure 4:**
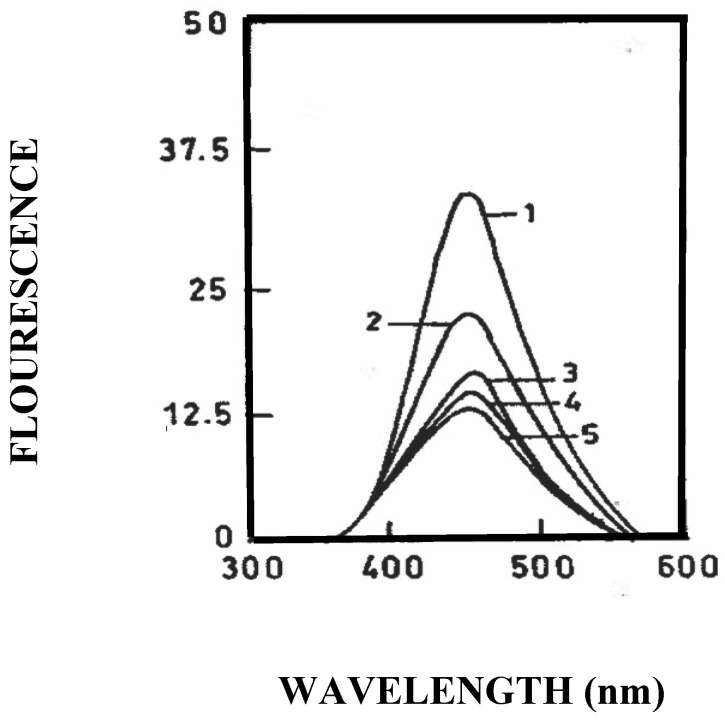
Fluorescence emission spectra of resveratrol at 25 °C. Resveratrol (in 5 mM phosphate buffer, pH 7.0), was excited at its λ_max_ (adsorption) of 308 and the emission spectra were recorded between 360 and 580 nm. Trace 1: resveratrol alone (5 µM); Trace 2: resveratrol: caprine casein (α_s1_-II) molar ratio 1:1; Trace 3: resveratrol: caprine casein (α_s1_-II) molar ratio 1:10; Trace 4: resveratrol: caprine casein (α_s1_-II) molar ratio 1:20; Trace 5: resveratrol: caprine casein (α_s1_-II) molar ratio 1:30.

The effect of increasing molar ratios of caprine casein (α_s1_-II) on the fluorescence emission spectrum of resveratrol was studied and the results are shown in Figure 4. When excited at its adsorption maximum of 308 nm, resveratrol exhibited a fluorescence emission with a peak at around 459 nm. The addition of caprine casein (α_s1_-II) at molar ratios of 1, 10, 20, and 30 resulted in a dose-dependent quenching of the fluorescence peak of resveratrol, thus confirming the binding of caprine casein (α_s1_-II) to resveratrol. As reported with the bovine casein/resveratrol complex [37], the caprine casein (α_s1_-II)/resveratrol complex is likely to be stabilized by hydrophobic interactions and hydrogen bonds.

### 3.5. Effects of Polysorbate-20 on the Physicochemical Properties of Resveratrol-Loaded Nanoemulsions Stabilized by Bovine and Caprine Caseins

As noted above, the fluorescence and CD spectroscopy data, especially the CD data concerning complex formation between caprine β-casein and polysorbate-20, place this protein in a helical conformational niche. The far-UV results, like those from the fluorescence spectra, clearly indicate the compatibility of polysorbate-20’s fatty acid chain with the hydrophobicity of caprine casein. The fluorescence data of caprine casein α_s1_-II/polysorbate-20 complex presented in Figure 1, therefore, suggest the presence of a significant amount of hydrophobicity in caprine casein (α_s1_-II). This would argue for a significant amount of β-casein for the caprine casein (α_s1_-II) (Table 1). Indeed, the β-casein content of the caprine caseins used in this study varies from 51.6% for the caprine casein (α_s1_-I) to 60.6% for the caprine casein (α_s1_-II) and, therefore, polysorbate-20 interacts with caprine casein (α_s1_-II) through hydrophobic interactions.

The hydrophilic and hydrophobic groups in various amino acid sequences and proportions make the bovine casein a candidate with good solubility, which is suitable for food emulsions and as a carrier of bioactive ingredients [38,39]. In bovine milk, casein assembles into micelles consisting of four proteins (α_s1_-, α_s2_-, β-, and κ-caseins) via hydrophobic interactions between the four individual caseins and calcium phosphate-mediated salt bridges, and is stabilized by the negative charge and steric repulsion of κ-casein [40]. The surface-active and emulsifying properties of bovine casein micelles have been compared to those of sodium caseinate [41]. Interfacial tension results showed that caseinate is more surface active than casein micelles at the oil-in-water interface [42]. On the other hand, polysorbate-20 stabilizes emulsions principally through steric repulsion and hence the addition of polysorbate-20 improves the role of sodium caseinate at the oil-in-water interface in low-pH emulsions [13].

Table 3 shows the mean particle size of the oil droplets in resveratrol-loaded nanoemulsions prepared with bovine and caprine caseins individually and in combination with polysorbate-20 as emulsifiers (stabilizers) at 0 h. Neither an increase in particle size nor creaming was observed in all of resveratrol-loaded nanoemulsions during 48-h of storage at 25 °C (data not shown). It should be noted here that the bovine and caprine caseins of Table 1 were treated with sodium hydroxide before being dried into powders, as described in the Materials and Methods. Therefore, the term casein rather than sodium caseinate is being used in this study. The mean particle range (205.84 ± 1.57 nm–191.09 ± 2.27 nm) of the oil droplets confirmed the suitability of all emulsifying (stabilizing) compounds in the preparation of the resveratrol-loaded emulsions. The interfacial behavior of the emulsifiers may contribute to the differences in droplet sizes [43,44].

Polysorbate-20 is a non-ionic emulsifier with much lower surface tension than water, showing low interfacial tension when mixed with the organic phase, which favors the formation of small droplets [44,45]; therefore, the combination of this compound with caprine casein produced emulsions with smaller (*p* < 0.05) particle sizes than using caprine casein individually (Table 3). It can be concluded that, among the emulsifiers (stabilizers), the combination of caprine casein and polysorbate-20 was the best choice in terms of mean particle size of emulsions. The existence of small oil droplets in the resveratrol-loaded nanoemulsions stabilized by the caprine casein/polysorbate-20 complex suggests that these oil droplets could be effectively taken up by the enterocytes for the improved bioavailability of the bound resveratrol. Leaving phytochemicals in a dispersed state make them more readily absorbed [46]. The delivery system of the present study is MCT oil-based, which diffuses passively from the gastrointestinal tract into the bloodstream, securing a superb bioavailability and a long-lasting effect [46].

All resveratrol-loaded nanoemulsions showed a negative surface charge (zeta potential), and the mixture of either bovine casein or caprine casein with polysorbate-20 produced resveratrol-loaded nanoemulsions with a low net surface charge (Table 3). At the pH used in this study (pH 7.0), the bovine and caprine caseins exhibited a negative charge. The charged groups of interfacial proteins present a barrier to the close approach and coalescence of neighboring droplets [43]. Moreover, the adsorption of OH groups from resveratrol onto the oil–water interface could also be responsible for the observed negative surface charges. The zeta potentials of the caprine casein-stabilized oil droplets in resveratrol-loaded nanoemulsions were not significantly (*p* > 0.05) affected by the addition of polysorbate-20, and exhibited lower zeta potential values compared with bovine casein in the presence of polysorbate-20 (Table 3). The lower phosphoserine contents of the caprine caseins yield lower zeta potentials [27], but the zeta potentials of the oil droplets were high enough to confer electrostatic repulsive forces.

### 3.6. Stability of Resveratrol

Emulsions are colloidal systems consisting of three components: the dispersed oil phase, also referred to as oil droplets, the continuous phase, and the oil–water interface stabilized by emulsifiers. The partition of resveratrol and surfactant molecules among the three different components depends on their solubility properties, surface activity, and affinity for the emulsifier at the oil–water interface [42]. The displacement of bovine casein or caprine casein from the oil–water interface did not occur at low polysorbate-20 content (≤1000 µM). Therefore, all of the polysorbate-20 interacted with bovine casein or caprine casein without affecting the fraction of milk proteins associated to the oil droplets. Moreover, the resveratrol isomer forms *trans*-resveratrol and *cis*-resveratrol were not detected in the continuous phase during processing/storage.

Chemical stability refers to how readily the resveratrol molecule can undergo chemical reactions that modify the hydroxyls and the carbon–carbon double bond [47,48]. The RP-HPLC results indicated that resveratrol degradation occurred in all prepared nanoemulsions due to the high sensitivity of the resveratrol chemical structure to thermal and oxidative stresses during the processing steps and the short-term storage at 25 °C (Table 4). The degradation of resveratrol in nanoemulsions during processing/storage might be seriously accelerated by the large total surface area of all oil droplets as a result of a size reduction to the nanometer scale [48]. The loss of resveratrol during processing/storage has been attributed to factors such as pH, heat, light, and oxygen [48]. In the present work, the chemical stability of resveratrol was strongly influenced by the emulsifiers used. Thus, caprine casein/polysorbate-20 complex appears to be more effective as an emulsifier than bovine casein/polysorbate-20 complex, and may therefore be the most suitable for stabilizing nanoemulsions that are loaded with resveratrol (Table 4). The role of the emulsifier on resveratrol stabilization appears to be due to the shielding of such hydrophobic phytochemical with a hydrophobic domain provided by a milk protein (e.g., caprine casein) via a surfactant (e.g., polysorbate-20).

**Figure 5:**
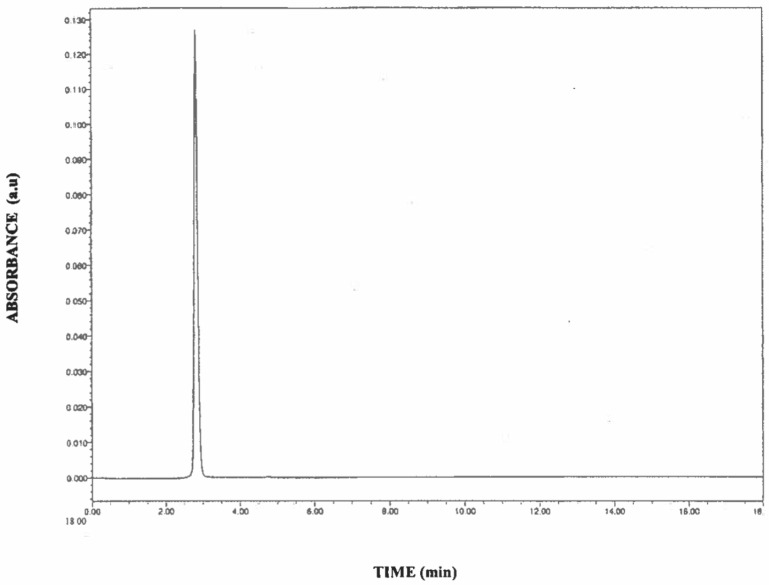
RP-HPLC chromatogram of *trans*-resveratrol.

It appears that resveratrol loses persistency in nanoemulsions with time, regardless of the type of emulsifier used (Table 4). However, the mixture of caprine casein (α_s1_-I) or caprine casein (α_s1_-II) with polysorbate-20 showed the least degradation of resveratrol (Table 4) during processing/storage as quantified by RP-HPLC. The chromatogram obtained at 306 nm was used for quantitative analysis of *trans*-resveratrol (Figure 5). Based on the retention time and intensity of the standard, the persistence of resveratrol in nanoemulsions was quantified at 6, 12, 24, and 48 h. Significant differences (*p* < 0.05) were observed between the nanoemulsions at 6 h and the nanoemulsions stored at 12, 24, and 48 h (at 25 °C). The mean persistence of resveratrol in nanoemulsions stabilized by caprine casein (α_s1_-I)/polysorbate-20 complex was 88.33 ± 1.84% during short-term storage at 25 °C for 48 h. Meanwhile, the mean persistence of resveratrol in caprine casein (α_s1_-II)/polysorbate-20 complex was 89.08 ± 2.14% during short-term storage at 25 °C for 48 h. Resveratrol was significantly (*p* < 0.05) less stable in nanoemulsions prepared with caprine casein (α_s1_-I) or caprine casein (α_s1_-II) in the absence of polysorbate-20 (Table 4). When tested for its ability to minimize the degradation of resveratrol, the bovine casein was found to produce the worst results. The differences in resveratrol degradation among the three caseins appeared to be related to the differences in β-casein content (Table 1).

Denaturation or unfolding is a general phenomenon specific for globular proteins. Proteins fold in a manner that minimizes the exposure of hydrophobic side chains of the protein to water [49]. The structure that the protein assumes depends on its surrounding environment (buffer solution, storage containers, etc.). In addition, there are intrinsic properties embedded in the primary amino acid sequence that may determine the degree of stability and hence the shelf life of protein products (e.g., protein-stabilized emulsions) [49]. The secondary structure of caprine β-casein was affected by polysorbate-20, as indicated by the CD spectra (Figure 3). It could be argued the correlation of modified spectra with structural changes is dominated by the interaction upon binding to polysorbate-20, thereby contributing to the pronounced negative ellipticity (Figure 3). The laureate chains of polysorbate-20 associate with disordered regions of casein, promoting the hydrophobic environment [35], which may stabilize the disordered structure and, thereby inducing α-helix and sheet conformation (Table 2). The data of Table 2 clearly suggest that polysorbate-20 protects caprine β-casein from aggregation due to association with disordered regions. Thus, for the better protection of resveratrol in nanoemulsion form, a mixture of caprine casein and polysorbate-20 as stabilizer is a good option.

Moreover, a significant (*p* < 0.05) initial decrease in resveratrol content in the nanoemulsions was observed overtime (Table 4). At 6-h oxidation, the mean persistence of resveratrol was 92.11 ± 0.99% for the bovine and caprine caseins individually and in combination with polysorbate-20. At 48 h oxidation, the mean persistence of resveratrol was only 76.17 ± 1.71% and no differences (*p* > 0.05) were found at 24 and 48 h in the nanoemulsions during a short-term storage at 25 °C for 48 h. These results suggest that the loss of resveratrol was fast at the oil–water interface, regardless of the surfactant and protein used. Around 500 µg/mL of the added resveratrol was bound to the bovine and caprine caseins, as described in the Materials and Methods. The binding is mainly governed by hydrophobic interactions close to the tryptophan residues [38]. Caprine caseins, which show higher hydrophobic character due to their high content of β-casein (Table 1), interact strongly with the resveratrol molecule (Figure 4). Conformational changes in caprine β-casein caused by the binding of polysorbate-20 can also expose or form hydrophobic environments on the caprine casein (α_s1_-I) and caprine casein (α_s1_-II). Therefore, the binding of hydrophobic resveratrol to the caprine casein/polysorbate-20 complex is likely to be driven by interaction with caprine β-casein. A recent study showed that resveratrol was less susceptible to degradation when entrapped in A2 β-casein from caprine milk and chitosan oligosaccharide [50]. In agreement with these findings, the results of our study implied a possible correlation between caprine β-casein functionality and the stability of resveratrol.

## 4. Conclusions

A fundamental understanding of the molecular details of the stability of β-casein will enable us to offer a sound explanation regarding the environmental factors (e.g., pH, heat, light, oxygen) that influence the chemical stability of resveratrol entrapped within a milk protein/surfactant matrix in nanoemulsion delivery systems. The results showed that nanoemulsion delivery systems stabilized by caprine casein and polysorbate-20 could slow down the degradation of the unstable resveratrol. Further investigations to characterize the chemical degradation of resveratrol in nanoemulsion delivery systems stabilized by emulsifiers (caprine casein, polysorbate-20) and hydrocolloids (polysaccharides) are greatly needed; these can modify the structure and composition of the adsorbed stabilizing layer, with important implications for texture and shelf life.

## Figures and Tables

**Table 1:** Casein distribution of caprine caseins compared with a typical bovine casein ^1^.

Casein Fraction %					
Sample	α_s2_	α_s1_	α_s1_	β	κ
Caprine casein high in α_s1_-casein-I	9.2	4.0	21.1	51.6	13.8
Caprine casein high in α_s1_-casein-II	5.3		25.6	60.6	9.6
Bovine casein	12.1		39.5	37.2	11.2

^1^ From Mora-Gutierrez et al. [28].

**Table 2:** Secondary structure of bovine and caprine β-caseins in the presence or in the absence of polysorbate-20.

β-Casein/Polysorbate-20 Samples				
Conformational Element *	Bovine β-Casein	Bovine β-Casein/PS-20 ^1^	Caprine β-Casein	Caprine β-Casein/PS-20
α-helix (%)	13.5	16.8	14.2	17.8
β-strand (%)	17.9	21.0	16.8	22.5
Irregular (%)	69.1	62.7	70.3	61.6

^1^ PS-20, polysorbate-20. * Analysis of secondary structure of β-casein/PS-20 samples was processed by the CAPITO program [20].

**Table 3:** Particle size and zeta potential of resveratrol-loaded nanoemulsions stabilized with surfactant and protein (emulsifiers).

Emulsifier	Particle Size (nm) ^1^	Zeta Potential (mV) ^1^
Bovine casein	205.84 ± 1.57 ^a^	−38.23 ± 0.29 ^c^
Bovine casein + PS-20 ^2^	202.89 ± 1.66 ^a^	−37.39 ± 1.56 ^b,c^
Caprine casein (α_s1_-I)	200.80 ± 0.45 ^a^	−34.95 ± 0.18 ^a^
Caprine casein (α_s1_-I) + PS-20	192.17 ± 4.61 ^b^	−33.74 ± 0.95 ^a^
Caprine casein (α_s1_-II)	200.16 ± 2.54 ^a^	−35.10 ± 0.69 ^b^
Caprine casein (α_s1_-II) + PS-20	191.09 ± 2.27 ^b^	−33.89 ± 1.19 ^a^

^1^ Mean value ± standard error. ^2^ PS-20, polysorbate-20. ^a–c^ Means in the same column with different superscripts are significantly different (*p* < 0.05).

**Table 4:** Persistence of resveratrol in nanoemulsions stabilized with surfactant and protein (emulsifiers) during storage for 48 h at 25 °C.

	Persistence of Resveratrol (%) ^2^
Emulsifier ^1^	
Bovine casein	76.25 ± 2.67 ^c^
Bovine casein + PS-20 ^2^	78.83 ± 2.61 ^c^
Caprine casein (α_s1_-I)	83.58 ± 1.90 ^b^
Caprine casein (α_s1_-I) + PS-20	88.33 ± 1.84 ^a^
Caprine casein (α_s1_-II)	83.50 ± 1.64 ^b^
Caprine casein (α_s1_-II) + PS-20	89.08 ± 2.14 ^a^
Storage time (hours)	
6	92.11 ± 0.99 ^a^
12	86.72 ± 1.11 ^b^
24	78.06 ± 1.49 ^c^
48	76.17 ± 1.71 ^c^

^1^ Emulsifier data are the mean of samples for each of the time periods studied (6, 12, 24, and 48 h); PS-20 = polysorbate-20. ^2^ Mean value ± standard error. ^a–c^ Means within each treatment with a different superscript are significantly different (*p* < 0.05).
